# The Association between Environmental Factors, Race, and Cognitive Status

**DOI:** 10.1093/geroni/igaa057.3296

**Published:** 2020-12-16

**Authors:** Ethan Siu Leung Cheung, Ada Mui

**Affiliations:** Columbia University, New York, New York, United States

## Abstract

Based on the data from National Social Life, Health and Aging Project, Wave 3, this study examined two research questions: what is the role of race in predicting cognitive status? and what are predictors of cognitive status between white and black older adults? Cognitive status was assessed using the 18-item survey-adapted Montreal Cognitive Assessment. Using the ecological framework, correlates of cognitive status were conceptualized in three levels of environments: micro- (personal health), meso- (social relationship), and macro-environments (community characteristics). Hierarchical regressions analyses were employed. Findings indicated that 83% of the sample (n= 2,829) were whites and the mean age was 72.95. Bivariate analyses suggested significant racial differences in cognitive status, marital status, income, education, health, social relationship, and community characteristics. Additive and interactive models showed that race had an independent effect as well as joint effects with the three levels of environments in explaining cognitive status. Parallel regression analyses for each racial group were undertaken and models were significant (P < .0001). In two separate models, common predictors for better cognition included being younger, more educated, fewer IADL impairments, and less depression. For older whites, unique correlates for better cognition were being female, higher income, sense of control in life, safer community, and neighbor relations. The only unique correlate for older blacks to have better cognition was community cohesion. Results provided insights on racial differences in cognition experienced among community-dwelling older Americans, and emphasized the need for social programs that promote race-sensitive, age-friendly communities to protect against cognitive decline.

